# The Invisible Toll: Unveiling the Prevalence and Predictors of Depression and Anxiety Among Pulmonary Tuberculosis (TB) Patients and Their Households in Gujarat, India

**DOI:** 10.7759/cureus.65015

**Published:** 2024-07-20

**Authors:** Nirmal Patel, Harita Patel, Jay Varu, Rohankumar Gandhi, Yogesh Murugan

**Affiliations:** 1 Preventive Medicine, Guru Govind Sinh Government Hospital, Jamnagar, IND; 2 Neuro-psychiatry, Gujarat Medical Education and Research Society Medical College, Valsad, Valsad, IND; 3 Internal Medicine, Shri Meghaji Pethraj Shah Government Medical College, Jamnagar, IND; 4 Community and Family Medicine, Shri Meghaji Pethraj Shah Government Medical College, Jamnagar, IND; 5 Family Medicine, Guru Govind Sinh Government Hospital, Jamnagar, IND

**Keywords:** stigma, social support, household contacts, anxiety, depression, tuberculosis

## Abstract

Background: Tuberculosis (TB) imposes a substantial physical and psychological burden on patients and their families. This study aimed to investigate the prevalence and predictors of depression and anxiety among pulmonary TB patients and their household contacts in Jamnagar, Gujarat, India.

Materials and methods: A cross-sectional study was conducted at TB units (TUs) in Jamnagar, Gujarat. Trained research assistants interviewed 272 pulmonary TB patients and 544 household contacts using structured questionnaires. Depression and anxiety were assessed using the Patient Health Questionnaire-9 (PHQ-9) and Hamilton Anxiety Rating Scale (HAM-A), respectively. Sociodemographic, clinical, and psychosocial factors (stigma and social support) were evaluated. Logistic regression analyses were performed to identify predictors of depression and anxiety. A p-value of < 0.05 was considered statistically significant for all analyses in this study.

Results: Out of 272 TB patients and 544 household contacts, the prevalence of depression was 98 (36.0%) and 135 (24.8%) (p=0.001). Anxiety was present in 85 (31.3%) of TB patients and 112 (20.6%) of household contacts (p<0.001). For TB patients, low household income (AOR=2.1, 95% CI: 1.9-4.3), low social support (AOR=0.84, 95% CI: 0.6-0.9), and high perceived stigma (AOR=2.3, 95% CI: 1.3-4.5) were independently associated with depression. Among household contacts, similar factors were identified, including low household income (AOR=1.7, 95% CI: 1.6-2.9), low social support (AOR=0.88, 95% CI: 0.6-0.9), and high perceived stigma (AOR=1.80, 95% CI: 1.1-2.3).

Conclusion: Depression and anxiety are highly prevalent among pulmonary TB patients and their household contacts in Gujarat, India. Low socioeconomic status, lack of social support, and TB-related stigma emerged as significant predictors of these mental health conditions, underscoring the need for integrated, multidisciplinary interventions to address the psychological impact of TB on patients and their families.

## Introduction

Tuberculosis (TB), a formidable adversary in infectious diseases, has long shadowed global public health. While the medical community has made strides in combating the physical manifestations of this ancient scourge, the psychological toll it exacts on patients and their families remains largely uncharted territory. Depression and anxiety, insidious companions to TB, often go unnoticed, yet their impact can be profound, undermining treatment adherence, prolonging recovery, and diminishing quality of life [1,2].

Mortality and incidence of TB decreased across all age groups for both males and females over the period 1990-2019. The incidence and mortality were higher among males than females [3]. Gujarat, a state renowned for its cultural richness and economic prowess, finds itself grappling with the dual challenge of TB and its psychological ramifications. Despite concerted efforts to address the physical manifestations of TB, the mental health aspects have remained an often-neglected facet, obscured by the urgency of clinical interventions [4].

Previous studies have shed light on the elevated prevalence of depression and anxiety among TB patients, underscoring the intricate relationship between physical and mental well-being [5,6]. However, the general impact and stress associated with a (relatively) chronic disease for children and adolescents with TB may also contribute to mental ill-health and affect their socioeconomic trajectory [7].

This study sought to unravel the intricate tapestry of depression, anxiety, and TB in Gujarat, India, by exploring the prevalence of these conditions among pulmonary TB patients and their household contacts. Furthermore, it aimed to elucidate the sociodemographic, clinical, and psychosocial factors that predispose individuals to these mental health challenges, providing a nuanced understanding of the complex interplay between TB and psychological distress [8,9].

By unveiling the hidden burdens of depression and anxiety in this population, the study endeavored to illuminate a path toward comprehensive care, where the physical and mental aspects of TB are addressed in tandem. Ultimately, the findings of this research endeavor to catalyze a paradigm shift, ushering in a holistic approach to TB management that transcends the boundaries of traditional medical interventions and embraces the multifaceted needs of patients and their families [10].

## Materials and methods

Study design and setting

This was a cross-sectional analytical study conducted at 3 TB units (TUs) in Jamnagar co-operation (2 Urban + 1 Rural) in Gujarat, India. The study was carried out between 23 March 2023 and 23 March 2024.

Eligibility criteria

The study population consisted of pulmonary TB (PTB) patients and their household contacts residing in Gujarat, India. The inclusion criteria for the study required pulmonary TB patients to be aged 18 years or older, diagnosed with active TB, and receiving anti-TB treatment at participating centers. Additionally, household contacts had to be aged 18 years or older and reside in the same household as the enrolled TB patients. Exclusion criteria were applied to exclude patients with extrapulmonary TB or multi-drug-resistant TB, as well as patients or household contacts with cognitive impairments or those unable to provide informed consent. Furthermore, household contacts who were not residing with the TB patient during the current treatment episode were also excluded from the study.

Sample size and sampling technique

For this study, a non-probability sampling approach was employed to recruit the required sample of TB patients and their household contacts. Specifically, a combination of purposive and snowball sampling techniques was utilized.

In the initial stage, the TUs in Jamnagar, Gujarat, India, were purposefully selected. At selected treatment centers, TB patients meeting the inclusion criteria were purposefully recruited through the coordination of healthcare providers and staff.

A sample of 272 TB patients was enrolled in TUs at that time, all patients who met inclusion criteria were included, and a snowball sampling technique was employed to recruit their household contacts. Each enrolled TB patient was asked to provide information about their household members, and those who met the inclusion criteria were invited to participate in the study.

The target sample size for household contacts was set at 544, maintaining a 1:2 ratio with the TB patient sample. This ratio was chosen to ensure a larger sample of household contacts, providing greater statistical power to assess the mental health burden and associated factors specific to this group.

The decision to use non-probability sampling techniques was based on the following considerations - (1) Accessibility and feasibility: TB patients and their household contacts can be considered hard-to-reach populations, and non-probability sampling methods allowed for more efficient and targeted recruitment within the available resources and timeframe. (2) Exploratory nature of the study: As this was an exploratory study investigating the prevalence and predictors of depression and anxiety in this population, non-probability sampling provided a pragmatic approach to gather initial insights and generate hypotheses for future research.

Data collection

Trained research assistants conducted face-to-face interviews with the study participants using structured questionnaires. The following data were collected - (1) Sociodemographic and clinical data: Age, gender, marital status, education, employment, income, substance abuse, and TB treatment details (for patients); (2) Depression and anxiety assessment: Depression was assessed using the Patient Health Questionnaire-9 (PHQ-9). A score ≥10 was considered indicative of depression [11]. Anxiety was assessed using the Hamilton Anxiety Rating Scale (HAM-A). A score ≥10 was considered indicative of anxiety [12]; and (3) Social support and perceived stigma: Social support was measured using the Multidimensional Scale of Perceived Social Support (MSPSS) [13,14] while the study utilized the perceived TB stigma scale to evaluate how participants perceived stigma related to TB.

Operational Definition

Depression: Depression was defined as a PHQ-9 score of 10 or higher. The PHQ-9 is a widely used and validated self-report measure for assessing the presence and severity of depressive symptoms. It consists of nine items based on the DSM-IV criteria for major depressive disorder, with scores ranging from 0 to 27. A score of 10 or higher has been shown to have high sensitivity and specificity for detecting major depressive disorder in various populations [11].

Anxiety: Anxiety was defined as a HAM-A score of 10 or higher. The HAM-A is a clinician-rated scale widely used to assess the severity of anxiety symptoms. It consists of 14 items, each rated on a scale of 0 (not present) to 4 (severe), with a total score ranging from 0 to 56. A score of 10 or higher is generally considered indicative of clinically significant anxiety [12].

Social support: Social support was measured using the MSPSS, a 12-item self-report instrument that assesses perceived social support from three sources: family, friends, and significant others. The MSPSS has been widely used and validated across various populations and cultures, demonstrating good internal consistency, test-retest reliability, and construct validity [13,14].

Perceived stigma: Perceived stigma related to TB was assessed using the perceived TB stigma scale. This scale comprises 11 items, each rated on a 4-point Likert scale from 1 (strongly disagree) to 4 (strongly agree). Participants were categorized as perceiving stigma if their score equaled or exceeded the mean stigma score. The scale demonstrated good internal consistency in this study, with a Cronbach’s alpha of 0.81 [15].

Validity and reliability of scales

The PHQ-9, HAM-A, MSPSS, and perceived TB stigma scale are widely used and validated instruments for assessing depression, anxiety, social support, and perceived stigma, respectively. In this study, the internal consistency of these scales was evaluated using Cronbach’s alpha, and the results demonstrated good reliability (PHQ-9: α = 0.85, HAM-A: α = 0.89, MSPSS: α = 0.92, perceived TB stigma scale: α = 0.81).

Data quality assurance

*Measures Implemented to Ensure the Quality and Integrity of the Data Collected* 

Training of research assistants: Research assistants underwent rigorous training in data collection procedures, interviewing techniques, and ethical considerations to ensure standardized and consistent data collection.

Pilot testing: The questionnaires and data collection procedures were pilot-tested with a small subset of participants to identify and address any potential issues or ambiguities before the main data collection phase.

Data entry and validation: All data were double-entered into a secure database by trained data entry personnel. Automated range and consistency checks were performed to identify and resolve potential data entry errors.

Regular monitoring: The principal investigator and supervisors regularly monitored the data collection process, reviewed completed questionnaires, and provided feedback to research assistants to maintain quality standards.

Data security and confidentiality: All data were anonymized and stored securely, with access restricted to authorized personnel only. Appropriate measures were taken to protect the confidentiality of participants’ information throughout the study.

Ethical considerations

The study protocol was approved by the Institutional Review Board/Ethics Committee (Protocol no: 257/03/2023) (M.P. Shah Medical College and Guru Gobind Singh Hospital Jamnagar). Written informed consent was obtained from all participants before enrollment. Confidentiality and privacy were maintained throughout the study.

Statistical analysis

Descriptive statistics were used to summarize the sociodemographic and clinical characteristics of the study participants. The prevalence of depression and anxiety was calculated as percentages with 95% CIs. Bivariate and multivariate logistic regression analyses were performed to identify factors associated with depression and anxiety among TB patients and household contacts. Odds ratios (ORs) with 95% CIs were calculated. A p-value <0.05 was considered statistically significant.

## Results

The sociodemographic characteristics of the study participants are presented in Table 1. This table provides a comprehensive overview of the demographic profile of both TB patients and their household contacts (HCC), which included 272 pulmonary TB patients and 544 HCC. The mean age of TB patients was 38.5 years (SD = 12.8), and for HCCs, it was 35.2 years (SD = 15.6). Among TB patients, 58.1% (n = 158) were male, and 41.9% (n = 114) were female, while in the HCC group, 53.1% (n = 289) were male, and 46.9% (n = 255) were female. The majority of TB patients (68.0%, n = 185) and HCCs (64.2%, n = 349) were married. In terms of education level, the highest proportion of TB patients (37.5%, n = 102) and HCCs (35.5%, n = 193) had completed primary education. Most TB patients (68.0%, n = 185) and HCCs (71.5%, n = 389) were employed. Regarding monthly household income, 45.6% (n = 124) of TB patients and 47.2% (n = 257) of HCCs had an income between 10,000 and 20,000 Indian rupees (INR). Substance abuse was reported by 23.9% (n = 65) of TB patients and 16.4% (n = 89) of HCCs. The mean social support score was 22.8 (SD = 6.2) for TB patients and 25.1 (SD = 7.4) for HCCs, while the mean perceived stigma score was 18.4 (SD = 5.8) for TB patients and 14.2 (SD = 6.1) for HCCs. Overall, these tables provide valuable insights into the prevalence of depression and anxiety among pulmonary TB patients and their household contacts, as well as the factors associated with these mental health conditions. The findings highlight the importance of addressing mental health issues and providing appropriate support to TB patients and their families.

**Table 1:** Sociodemographic Characteristics of the Study Participants INR: Indian Rupees

Characteristic	TB Patients (n=272)	HCC (n=544)
Age, mean (SD), years	38.5 (12.8)	35.2 (15.6)
Gender, n (%)		
Male	158 (58.1)	289 (53.1)
Female	114 (41.9)	255 (46.9)
Marital Status, n (%)		
Unmarried	67 (24.6)	152 (27.9)
Married	185 (68.0)	349 (64.2)
Widowed/divorced	20 (7.4)	43 (7.9)
Education Level, n (%)		
No formal education	45 (16.5)	68 (12.5)
Primary	102 (37.5)	189 (34.7)
Secondary	87 (32.0)	193 (35.5)
Higher secondary and above	38 (14.0)	94 (17.3)
Employment Status, n (%)		
Employed	185 (68.0)	389 (71.5)
Unemployed	87 (32.0)	155 (28.5)
Monthly Household Income, n (%)		
< 10,000 INR	98 (36.0)	172 (31.6)
10,000-20,000 INR	124 (45.6)	257 (47.2)
> 20,000 INR	50 (18.4)	115 (21.1)
Substance abuse, n (%)	65 (23.9)	89 (16.4)
Social support score, mean (SD)	22.8 (6.2)	25.1 (7.4)
Perceived stigma score, mean (SD)	18.4 (5.8)	14.2 (6.1)

Table 2 shows the prevalence of depression and anxiety among pulmonary TB patients and their household contacts. Depression, defined as a PHQ-9 score of 10 or higher, was present in 36.0% (n=98) of TB patients and 24.8% (n=135) of HCCs. The prevalence of depression was significantly higher among TB patients compared to HCCs (p=0.001). Similarly, anxiety, defined as a HAM-A score of 10 or higher, was present in 31.3% (n=85) of TB patients and 20.6% (n=112) of HCCs. The prevalence of anxiety was also significantly higher among TB patients compared to HCCs (p<0.001).

**Table 2:** Prevalence of Depression and Anxiety among Pulmonary TB Patients and Their Household Contacts PHQ-9: Patient Health Questionnaire-9; HAM-A: Hamilton Anxiety Rating Scale p<0.05*: significant, p<0.001**: highly significant

Condition	TB Patients (n=272)	Household Contacts (n=544)	p-value
Depression (PHQ-9 ≥ 10), n (%)	98 (36.0)	135 (24.8)	<0.001 **
Anxiety (HAM-A ≥ 10), n (%)	85 (31.3)	112 (20.6)	<0.001 **

Table 3 presents the results of bivariate logistic regression analyses examining the association between various factors and depression/anxiety among TB patients and HCCs. Among TB patients, factors significantly associated with higher odds of depression included female gender (COR=1.8, 95% CI: 1.1-3.0), low education level (no formal education: COR=2.5, 95% CI: 1.2-5.1; primary: COR=1.8, 95% CI: 1.0-3.2), unemployment (COR=1.7, 95% CI: 1.0-2.9), low household income (<10,000 INR: COR=2.8, 95% CI: 1.5-5.2; 10,000-20,000 INR: COR=1.9, 95% CI: 1.1-3.3), substance abuse (COR=2.1, 95% CI: 1.2-3.7), low social support (COR=0.7, 95% CI: 0.6-0.9), and high perceived stigma (COR=2.5, 95% CI: 1.8-7.1). Similar patterns were observed for the association between these factors and anxiety among TB patients. Among HCCs, factors significantly associated with higher odds of depression included female gender (COR=1.5, 95% CI: 1.0-2.2), low education level (no formal education: COR=1.9, 95% CI: 1.1-3.4; primary: COR=1.5, 95% CI: 1.0-2.3), low household income (<10,000 INR: COR=2.1, 95% CI: 1.3-3.4; 10,000-20,000 INR: COR=1.6, 95% CI: 1.1-2.4), substance abuse (COR=1.7, 95% CI: 1.1-2.7), low social support (COR=0.8, 95% CI: 0.7-0.9), and high perceived stigma (COR=2.0, 95% CI: 1.3-5.1). Similar patterns were observed for the association between these factors and anxiety among HCCs, although some associations were not statistically significant.

**Table 3:** Bivariate Analysis of Factors Associated with Depression and Anxiety P<0.05*: significant; P<0.001**: highly significant; COR: crude odds ratio

Characteristic	TB Patients (n=272)		Household Contacts (n=544)	
	Depression	Anxiety	Depression	Anxiety
	COR (95% CI)	COR (95% CI)	COR (95% CI)	COR (95% CI)
Age (Years)				
≥ 40	1.2 (0.7-2.1)	1.4 (0.8-2.4)	1.1 (0.7-1.7)	1.3 (0.8-2.0)
< 40	Reference	Reference	Reference	Reference
Gender				
Female	1.8 (1.1-3.0)*	1.6 (0.9-2.7)	1.5 (1.0-2.2)*	1.4 (0.9-2.1)
Male	Reference	Reference	Reference	Reference
Marital Status				
Unmarried	1.5 (0.8-2.8)	1.2 (0.6-2.3)	1.3 (0.8-2.1)	1.1 (0.7-1.8)
Widowed/divorced	2.1 (0.9-4.9)	1.8 (0.7-4.4)	1.7 (0.9-3.2)	1.5 (0.8-3.0)
Married	Reference	Reference	Reference	Reference
Education Level				
No formal education	2.5 (1.2-5.1)*	2.1 (1.0-4.5)*	1.9 (1.1-3.4)*	1.6 (0.9-2.9)
Primary	1.8 (1.0-3.2)*	1.6 (0.9-2.9)	1.5 (1.0-2.3)*	1.3 (0.8-2.0)
Secondary	1.3 (0.7-2.4)	1.1 (0.6-2.1)	1.2 (0.8-1.8)	1.0 (0.6-1.6)
Higher secondary and above	Reference	Reference	Reference	Reference
Employment Status				
Unemployed	1.7 (1.0-2.9)*	1.5 (0.9-2.6)	1.4 (0.9-2.1)	1.2 (0.8-1.9)
Employed	Reference	Reference	Reference	Reference
Monthly Household Income				
< 10,000 INR	2.8 (1.5-5.2)**	2.4 (1.3-4.5)**	2.1 (1.3-3.4)**	1.8 (1.1-2.9)*
10,000-20,000 INR	1.9 (1.1-3.3)*	1.7 (1.0-3.0)*	1.6 (1.1-2.4)*	1.4 (0.9-2.1)
> 20,000 INR	Reference	Reference	Reference	Reference
Substance Abuse				
Yes	2.1 (1.2-3.7)*	1.9 (1.1-3.4)*	1.7 (1.1-2.7)*	1.5 (0.9-2.4)
No	Reference	Reference	Reference	Reference
Social support score	0.7 (0.6-0.9)**	0.81 (0.7-0.9)**	0.8 (0.7-0.9)*	0.72 (0.5-0.8)*
Perceived stigma score	2.5 (1.8-7.1)**	2.2 (1.2-5.1)**	2.0 (1.3-5.1)**	3.3 (2.08-6)

Table 4 presents the results of multivariate logistic regression analyses, which examined the independent association between various factors and depression/anxiety, adjusting for potential confounders. Among TB patients, low household income (<10,000 INR: AOR=2.1, 95% CI: 1.9-4.3), low social support (AOR=0.84, 95% CI: 0.6-0.9), and high perceived stigma (AOR=2.3, 95% CI: 1.3-4.5) were independently associated with higher odds of depression after adjusting for other factors. For anxiety among TB patients, low social support (AOR=0.87, 95% CI: 0.8-0.9) and high perceived stigma (AOR=2.09, 95% CI: 1.4-5.1) remained independently associated.

**Table 4:** Multivariate Logistic Regression Analysis of Factors Associated with Depression and Anxiety P<0.05*: significant; P<0.001**: highly significant; AOR: adjusted odds ratio

Characteristic	TB Patients (n=272)		Household Contacts (n=544)	
	Depression	Anxiety	Depression	Anxiety
	AOR (95% CI)	AOR (95% CI)	AOR (95% CI)	AOR (95% CI)
Age (Years)				
≥ 40	1.1 (0.6-2.0)	1.3 (0.7-2.3)	1.0 (0.6-1.6)	1.2 (0.7-1.9)
< 40	Reference	Reference	Reference	Reference
Gender				
Female	1.6 (0.9-2.8)	1.4 (0.8-2.5)	1.4 (0.9-2.1)	1.3 (0.8-2.0)
Male	Reference	Reference	Reference	Reference
Education Level				
No formal education	2.0 (0.9-4.4)	1.8 (0.8-4.0)	1.6 (0.9-3.0)	1.4 (0.7-2.7)
Primary	1.5 (0.8-2.8)	1.4 (0.7-2.6)	1.3 (0.8-2.1)	1.2 (0.7-1.9)
Secondary	1.2 (0.6-2.3)	1.0 (0.5-2.0)	1.1 (0.7-1.7)	0.9 (0.6-1.5)
Higher secondary and above	Reference	Reference	Reference	Reference
Employment Status				
Unemployed	1.4 (0.8-2.5)	1.3 (0.7-2.3)	1.2 (0.8-1.9)	1.1 (0.7-1.7)
Employed	Reference	Reference	Reference	Reference
Monthly Household Income				
< 10,000 INR	2.1 (1.9-4.3)*	1.9 (0.9-3.9)	1.7 (1.6-2.9)*	1.5 (0.9-2.6)
10,000-20,000 INR	1.6 (0.9-2.9)	1.5 (0.8-2.7)	1.4 (0.9-2.2)	1.3 (0.8-2.0)
> 20,000 INR	Reference	Reference	Reference	Reference
Substance Abuse				
Yes	1.7 (0.9-3.1)	1.6 (0.9-3.0)	1.5 (0.9-2.4)	1.4 (0.8-2.3)
No	Reference	Reference	Reference	Reference
Social support score	0.84 (0.6-0.9)**	0.87 (0.8-0.9)**	0.88 (0.6-0.9)*	0.79 (0.66-0.89)*
Perceived stigma score	2.3 (1.3-4.5)**	2.09 (1.4-5.1)**	1.80 (1.1-2.3)**	2.87 (1.90-7)**

Among HCCs, low household income (<10,000 INR: AOR=1.7, 95% CI: 1.6-2.9), low social support (AOR=0.88, 95% CI: 0.6-0.9), and high perceived stigma (AOR=1.80, 95% CI: 1.1-2.3) were independently associated with higher odds of depression after adjusting for other factors. For anxiety among HCCs, low social support (AOR=0.79, 95% CI: 0.66-0.89) and high perceived stigma (AOR=2.87, 95% CI: 1.90-7) remained independently associated.

Overall, these tables provide valuable insights into the prevalence of depression and anxiety among pulmonary TB patients and their household contacts, as well as the factors associated with these mental health conditions. The findings highlight the importance of addressing mental health issues and providing appropriate support to TB patients and their families.

## Discussion

This study aimed to investigate the prevalence and predictors of depression and anxiety among pulmonary TB patients and their household contacts in Jamnagar, Gujarat, India. The findings revealed a high burden of mental health issues in this population, with a significant proportion of TB patients and their household contacts experiencing depression and anxiety.

Prevalence of depression and anxiety

The prevalence of depression among TB patients (36.0%) and their household contacts (24.8%) observed in this study is consistent with previous reports from similar settings. A prospective study by Ambaw et al. found that the prevalence of probable depression and suicidal ideation among TB patients was 53.9% and 17.4% [16]. Similarly, a meta-analysis also reported a prevalence of depression among TB patients of 45.19% (95% CI 38.04-52.55) [17]. The high prevalence of depression in our study underscores the need for integrating mental health screening and support services into TB care programs.

The prevalence of anxiety among TB patients (31.3%) and their household contacts (20.6%) in our study is also aligned with previous findings. A systematic review also found that the pooled prevalence of anxiety among TB patients was 32.54% (24.95, 41.18) [18]. The high rates of anxiety observed in our study population highlight the psychological distress and emotional burden associated with TB and its impact on family members.

Factors associated with depression and anxiety

The bivariate and multivariate analyses identified several sociodemographic, clinical, and psychosocial factors associated with depression and anxiety among TB patients and their household contacts. Low household income, low social support, and high perceived stigma emerged as independent predictors of depression in both groups, after adjusting for potential confounders. These findings are consistent with previous studies that have reported the adverse impact of poverty, a lack of social support, and TB-related stigma on mental health outcomes [19-21].

Additionally, substance abuse was found to be associated with increased odds of depression and anxiety among TB patients in the bivariate analysis, although this association did not remain significant in the multivariate model. This finding aligns with previous research suggesting a bidirectional relationship between substance abuse and mental health disorders, where individuals with mental health issues may resort to substance use as a coping mechanism, and substance abuse can exacerbate or contribute to the development of mental health problems [22].

Implications and recommendations

The high prevalence of depression and anxiety among TB patients and their household contacts, coupled with the identified risk factors, underscores the need for a comprehensive and integrated approach to TB care. Mental health screening and support services should be incorporated into TB treatment programs to address the psychological and emotional challenges faced by patients and their families.

Interventions aimed at reducing poverty, enhancing social support networks, and combating TB-related stigma may also play a crucial role in mitigating the risk of depression and anxiety in this population. Collaborative efforts involving healthcare providers, mental health professionals, community organizations, and policymakers are necessary to develop and implement effective strategies for addressing the mental health needs of TB patients and their household contacts.

Furthermore, targeted interventions focused on substance abuse prevention and treatment may be beneficial in reducing the burden of mental health disorders among TB patients. Integrating substance abuse counseling and rehabilitation services into TB care programs could potentially improve treatment adherence and overall health outcomes.

Strengths and limitations

One of the strengths of this study is its comprehensive assessment of both TB patients and their household contacts, providing insights into the mental health burden and associated factors within the broader family context. Additionally, the use of standardized and validated instruments for assessing depression, anxiety, social support, and perceived stigma enhances the reliability of the findings.

However, the study has some limitations. The cross-sectional design precludes the establishment of causal relationships between the identified factors and mental health outcomes. Longitudinal studies are warranted to better understand the temporal associations and potential bidirectional effects and also to better capture the long-term effects of such interventions on mental health outcomes in TB patients and their families. Furthermore, the findings may not be directly applicable to other regions or populations with different sociodemographic and cultural contexts. We have suggested that future multi-center or multi-region studies could help address this limitation and provide more broadly applicable results. We also acknowledge that our findings may not be generalizable to the important subgroups (like extrapulmonary TB, multi-drug-resistant TB, < 18 years of age, and those with cognitive impairments or unable to provide informed consent or prior mental health history and concurrent medical conditions). We have also suggested that future research should aim to include these populations to provide a more comprehensive understanding of mental health issues across all TB patient groups. We also acknowledge that the 2-week timeframe of the PHQ-9 may not fully capture the chronic nature of depression in TB patients and suggested that future studies could consider using additional or alternative measures that assess depressive symptoms over longer periods to better understand the chronic aspects of depression in this population. We also suggested that future studies could consider alternative methods of identifying and recruiting household contacts to minimize potential bias or incomplete information.

## Conclusions

This study highlights the high prevalence of depression and anxiety among pulmonary TB patients and their household contacts in Jamnagar, Gujarat, India. Low household income, low social support, and high perceived stigma emerged as significant predictors of these mental health conditions. The findings underscore the pressing need for integrating mental health screening and support services into TB care programs, as well as addressing the underlying socioeconomic and psychosocial factors that contribute to the burden of mental health disorders in this population.

## Appendices

Questionnaire

1 Age: _____ years

2. Gender: □ Male □ Female □ Other

3. Marital Status: □ Unmarried □ Married □ Widowed □ Divorced

4. Education Level: □ No formal education □ Primary □ Secondary □ Higher secondary and above

5. Employment Status: □ Employed □ Unemployed

6. Monthly Household Income: □ < 10,000 INR □ 10,000-20,000 INR □ > 20,000 INR

7. Do you use any substances (e.g., alcohol, tobacco, drugs)? □ Yes □ No

Patient Health Questionnaire-9 (PHQ-9)

Over the last 2 weeks, how often have you been bothered by any of the following problems?

(0 = Not at all, 1 = Several days, 2 = More than half the days, 3 = Nearly every day)

1. Little interest or pleasure in doing things

2. Feeling down, depressed, or hopeless

3. Trouble falling or staying asleep, or sleeping too much

4. Feeling tired or having little energy

5. Poor appetite or overeating

6. Feeling bad about yourself or that you are a failure or have let yourself or your family down

7. Trouble concentrating on things, such as reading the newspaper or watching television

8. Moving or speaking so slowly that other people could have noticed, or the opposite - being so fidgety or restless that you have been moving around a lot more than usual

9. Thoughts that you would be better off dead or of hurting yourself in some way

Hamilton Anxiety Rating Scale (HAM-A)

For each item, please indicate the extent to which you have experienced the following symptoms over the past week:

(0 = Not present, 1 = Mild, 2 = Moderate, 3 = Severe, 4 = Very severe)

1. Anxious mood

2. Tension

3. Fears

4. Insomnia

5. Intellectual (cognitive)

6. Depressed mood

7. Somatic (muscular)

8. Somatic (sensory)

9. Cardiovascular symptoms

10. Respiratory symptoms

11. Gastrointestinal symptoms

12. Genitourinary symptoms

13. Autonomic symptoms

14. Behavior at interview

Multidimensional Scale of Perceived Social Support (MSPSS)

Please indicate how you feel about each statement.

(1 = Very Strongly Disagree, 2 = Strongly Disagree, 3 = Mildly Disagree, 4 = Neutral, 5 = Mildly Agree, 6 = Strongly Agree, 7 = Very Strongly Agree)

1. There is a special person who is around when I am in need

2. There is a special person with whom I can share my joys and sorrows

3. My family really tries to help me

4. I get the emotional help and support I need from my family

5. I have a special person who is a real source of comfort to me

6. My friends really try to help me

7. I can count on my friends when things go wrong

8. I can talk about my problems with my family

9. I have friends with whom I can share my joys and sorrows

10. There is a special person in my life who cares about my feelings

11. My family is willing to help me make decisions

12. I can talk about my problems with my friends

Perceived TB Stigma Scale

Please indicate how much you agree or disagree with each statement:

(1 = Strongly Disagree, 2 = Disagree, 3 = Agree, 4 = Strongly Agree)

1. Some people may not want to eat or drink with friends who have TB

2. Some people feel uncomfortable about being near those with TB

3. If a person has TB, some community members will behave differently toward that person for the rest of their life

4. Some people do not want those with TB playing with their children

5. Some people keep their distance from people with TB

6. Some people think that those with TB are disgusting

7. Some people do not want to talk to others with TB

8. Some people are afraid of those with TB

9. Some people try not to touch others with TB

10. Some people may not want to eat or drink with relatives who have TB

11. Some people prefer not to have those with TB living in their community
